# Novel mechanism regulating endothelial permeability via T-cadherin-dependent VE-cadherin phosphorylation and clathrin-mediated endocytosis

**DOI:** 10.1007/s11010-013-1867-4

**Published:** 2013-10-18

**Authors:** Ekaterina V. Semina, Kseniya A. Rubina, Veronika Yu. Sysoeva, Pavel N. Rutkevich, Natalia M. Kashirina, Vsevolod A. Tkachuk

**Affiliations:** 1 Russian Cardiology Research-Industrial Complex, Ministry of Health, 3rd Cherepkovskaya St. 15a, 121552 Moscow, Russia; 2 Faculty of Basic Medicine, Lomonosov Moscow State University, Lomonosov Ave 31/5, 119192 Moscow, Russia

**Keywords:** T-cadherin, VE-cadherin, Endothelial permeability, Rho GTPases

## Abstract

T-cadherin is a unique member of the cadherin superfamily of adhesion molecules. In contrast to “classical” cadherins, T-cadherin lacks transmembrane and cytoplasmic domains and is anchored to the cell membrane via a glycosilphosphoinositol moiety. T-cadherin is predominantly expressed in cardiovascular system. Clinical and biochemical studies evidence that expression of T-cadherin increases in post-angioplasty restenosis and atherosclerotic lesions—conditions associated with endothelial dysfunction and pathological expression of adhesion molecules. Here, we provide data suggesting a new signaling mechanism by which T-cadherin regulates endothelial permeability. T-cadherin overexpression leads to VE-cadherin phosphorylation on Y731 (β-catenin-binding site), VE-cadherin clathrin-dependent endocytosis and its degradation in lysosomes. Moreover, T-cadherin overexpression results in activation of Rho GTPases signaling and actin stress fiber formation. Thus, T-cadherin up-regulation is involved in degradation of a key endothelial adhesion molecule, VE-cadherin, resulting in the disruption of endothelial barrier function. Our results point to the role of T-cadherin in regulation of endothelial permeability and its possible engagement in endothelial dysfunction.

**Electronic supplementary material:**

The online version of this article (doi:10.1007/s11010-013-1867-4) contains supplementary material, which is available to authorized users.

## Introduction

Endothelium is a key regulator of vascular homeostasis, since it performs a barrier function and responds to physical and chemical signals by producing a broad spectrum of factors that regulate vascular tone, cellular adhesion, smooth muscle cell migration and proliferation, vessel wall inflammation, and thrombogenicity [1]. Endothelial dysfunction manifests itself in various pathological conditions such as atherosclerosis, hypertension, and diabetes mellitus. More specifically, at the physiological level endothelial dysfunction is characterized by changes in endothelial phenotype and particularly by the expression pattern of adhesion molecules involved in the cell–cell adhesion and endothelial permeability [2]. VE-cadherin plays a crucial role in maintaining endothelial cell monolayer integrity and regulation of its activity/localization at the adherens junctions is under control of permeability regulating factors [3].

VE-cadherin mediates cell adhesion via homophilic Ca^2+^-dependent interaction between its extracellular domains on the neighboring cells. Similar to other “classical” cadherins, VE-cadherin interacts via its cytoplasmic tail with p120^ctn^, β-catenin, α-catenin, plakoglobin, and cytoskeleton; thus, mediating strong cell–cell adhesion [3–5]. Most of the conditions that increase endothelial permeability affect the organization of the cadherin–catenin complex leading to the cell retraction and/or formation of intracellular gaps [5]. Several publications indicate that there is a correlation between changes in the stability of VE-cadherin-mediated junctions and phosphorylation status of proteins of cadherin–catenin complex [3, 5].

As it has been found recently by us and others, another cadherin namely T-cadherin (T-cad) expressed in endothelial cells, can modulate endothelial permeability [6, 7]; however, the exact mechanisms of T-cad participation remain obscure. T-cad is a unique member of the cadherin superfamily of adhesion molecules mediating Ca^2+^-dependent cell–cell adhesion [8]. Unlike other cadherins, T-cad does not have either transmembrane or cytoplasmic parts and is anchored to the cell membrane via a glycosilphosphoinositol (GPI) moiety [9]. The lack of an intracellular domain in T-cad structure suggests that T-cad is involved in the intracellular signaling rather than in stable cell–cell adhesion. Indeed, it was demonstrated that plating of endothelial cell on the substratum containing recombinant T-cad results in the rearrangement of actin cytoskeleton and cell detachment mediated via activation of RhoA and Rac1 signaling [7, 10]. In the present study, we used endothelial cells and examined the responses of RhoA, Rac1, and Cdc42 and their downstream signaling adaptors, ROCK-II and PAK1, as well as the changes in organization of actin cytoskeleton to overexpression of T-cad or to suppression of its native expression.

In our laboratory, it was shown that T-cad is predominantly expressed in a cardiovascular system in endothelial cells, smooth muscle cells, pericytes and cardiomyocytes [11, 12]. Expression of T-cad increases in the cases of post-angioplasty restenosis and atherosclerotic lesions—conditions associated with the increased cell migration, endothelial dysfunction, and pathological expression of adhesion molecules [11, 12]. Moreover, it has been reported that T-cad plays an essential role in revascularization after experimental limb ischemia [13].

The above-mentioned data prompted us to ask a question if T-cad is involved in regulation of the endothelial barrier function and to explore the possible mechanisms for its participation. To perform this study, we developed lentiviral constructs for overexpression or suppression of T-cad in primary endothelial cells. It was found that the overexpression of T-cad caused the increase in permeability of endothelial monolayer to macromolecules, contrary to which the suppression of native T-cadherin (si-T-cad) resulted in reduced permeability. We assessed the localization of VE-cadherin and revealed the phosphorylation status of specific tyrosine residues of VE-cadherin in control, T-cad and si-T-cad cells. It was shown that T-cad overexpression induced VE-cadherin phosphorylation on Y731, but not on Y658, resulting in VE-cadherin clathrin-mediated endocytosis and degradation in lysosomes. Thus, we suggest the existence of a new biochemical pathway by which T-cad regulates endothelial permeability. We suppose that the upregulation of T-cad expression is an important factor under conditions characterized by vascular leakage.

## Materials and methods

### Cell culture and reagents

Human umbilical vein endothelial cells (HUVEC) of the 2nd passage (HUVEC, CC-2517, Lonza) were grown in EBM-2 media, supplemented with SingleQuots^®^ Kit (CC-4176, Lonza). Cells were maintained at 37 °C in a humidified atmosphere of 5 % CO_2_ and 95 % air. Cells were used at passages 0–4.

### Lentiviral constructs

The lentiviral T-cad expressing construct pSIH-H1-Tcad was prepared using a pSIH1-H1-puro lentiviral vector (System Bioscience), as described in supplementary materials. For RNA interference mediated T-cad gene silencing the lentiviral construct pSIH-H1-puro-siTcad was used, as described in supplementary materials. For control transduction we used the constructs described before [7]. HUVEC of the 2nd passage were transduced with pseudoviral particles for overexpression of T-cad or silencing (si-T-cad) or for control experiments (control). The T-cad expression was assessed by immunofluorescent staining and immunoblotting with antibodies against human T-cad 78 h after transduction. The data was normalized by the GAPDH content.

### Antibodies and reagents

The following antibodies were used: rabbit anti-T-cad (ProSci Inc.); mouse anti-VE-cadherin, rabbit anti-phospho VE-cadherin (Y658), and rabbit anti-phospho VE-cadherin (Y731) (Abcam); mouse anti-N-cadherin (Santa Cruz); mouse anti-β-catenin and mouse anti-phospho-β-catenin (T41) (Abcam); mouse anti-p120^ctn^ (Santa Cruz); rabbit anti-clathrin (Abcam); rabbit anti- PAK1 and rabbit anti-phospho-PAK1 (T212) (Abcam); rabbit anti-ROCK-II (Santa Cruz); rabbit anti-Src and rabbit anti-phospho-Src (Y418) (Abcam); mouse anti-p38 (Sigma) and rabbit anti-phospho-p38 (T180+Y182) (Abcam); rabbit anti-Erk1+Erk2 ant rabbit anti-phospho-Erk1+Erk2 (T185+T202) (Abcam); mouse anti-ZO-1 (Invitrogen); mouse anti-occludin (Abcam); mouse anti-claudin-5 (Invitrogen); mouse anti caveolin-1 (BD); rabbit anti-EEA1 (Abcam); rabbit anti-LAMP1 (Abcam); LysotrackerRed^®^ (Invitrogen); phalloidin AlexaFluor^®^488 (Molecular Probes); secondary anti-mouse and anti-rabbit AlexaFluor^®^488 or AlexaFluor^®^594 antibodies (Molecular Probes) were used in immunofluorescence techniques. Rabbit anti-GAPDH (Santa Cruz) antibody was used in Western blotting for protein loading control; the secondary antibodies in Western blotting were HRP-conjugated donkey anti-mouse or donkey anti-rabbit IgG (Jackson Immuno-Research Laboratories). Chemicals for Western blotting were from Bio-Rad, buffers for immunofluorescence were purchased from GIBCO. Trypsin and gelatin were purchased from Sigma.

### Western blotting

HUVEC grown to confluency were washed twice with ice-cold HBSS and lysed in 2× Laemmli’s sample buffer containing β-mercaptoethanol and Protease Inhibitor Cocktail (Sigma). After 10 min of incubation lysates were disrupted using a G21 syringe and heated for 10 min at 90 °C, electrophoresed on SDS-PAGE (7.5 or 14 %) gel and electroblotted onto nitrocellulose membranes (Immobilon, Millipore). Kaleidoscope Prestained Standards (Bio-Rad) were used as molecular weight markers. Membranes were pre-blocked for 30 min in blocking buffer, containing 5 % (w/v) of nonfat milk, 0.5 % Tween 20 in PBS and then incubated with primary and secondary antibodies for 1 h. The signal was developed using ECL™ Western Blotting Detection Reagents and a CL-Xposure™ Film (all from Amersham). All experiments were repeated at least three different times in triplicates. Densitometric analysis of blots was performed using GS-800 Calibrated Densitometer (Bio-Rad) and Quantity One 4.6 Software (Bio-Rad).

### Immunofluorescence and confocal imaging

HUVEC f the 2nd passage were placed on gelatin-coated glass coverslips in concentration 2 × 10^4^ cells/cm^2^ and grown to confluence. For intracellular marker staining cells were washed with warm HBSS, fixed with 4 % paraformaldehyde, permeabilized for 5 min with 0.2 % Triton X-100 (if unless otherwise indicated), and blocked with 10 % normal donkey serum with 1 % BSA. After sequential incubation with primary and secondary antibodies according to concentrations recommended by manufacturer, coverslips were mounted with ProLong^®^ Antifade reagent with DAPI (Invitrogen) for nuclei staining. In internalization assay HUVEC were preincubated with dynasore hydrate (Sigma) in final concentration 80 μM for 2 h, or LysoTracker^®^Red (Invitrogen, Ex/Em: 577/590 nm) for 60 and 120 min, or Y27632 in final concentration 10 μM (Sigma) for 12 h before immunofluorescent staining.

Images were acquired by confocal laser scanning microscopy using a microscope system (TCS SP5, Leica) equipped with a Plan-Apo ×60, 1.40 NA oil objective and 488- and 543- Argon lasers lines. Imaging was performed at room temperature using Leica Type F immersion oil. DAPI, AlexaFluor^®^488 and AlexaFluor^®^594 fluorescence was sequentially excited using lasers with wavelengths of 405 (DAPI), 488 (AlexaFluor^®^488), and 594 (AlexaFluor^®^594). All images were captured using the same confocal gain and offset settings. Images (1024 × 1024 or 2048 × 2048 pixels) were saved as TIFF files in Leica LAS software and then complied in Photoshop (version CS5, Adobe) to generate figures. Labeling detected in individual channels is presented in red and green and then merged in individual panels; overlap of red and green staining is revealed in yellow. Results of at least three independent experiments are presented. Co-localization was acquired by immunofluorescence confocal analysis and quantitated using the ImageJ co-localization plug-in.

### Isolation of active Rho GTPases (GST pull-down assay)

Active Rho GTPases (RhoA, Rac1, and Cdc42) were isolated using commercial RhoA/Rac1/Cdc42 activation Assay Combo kit (Cellbiolabs Inc.). Active RhoA, Rac1, and Cdc42 GTPases were determined by electrophoresis in 14 % polyacrylamide gel with subsequent immunoblotting in the presence of monoclonal antibodies specific for each GTPase. For the positive control commercial available lysates were used. The total level of GTPases was determined in the lysates prepared as described. Results of three independent experiments are presented.

### In vitro determination of endothelial monolayer permeability

The endothelial cells monolayer permeability was assessed as described earlier [7]. In brief, HUVEC of the 3rd passage were plated (1 × 10^5^ cells/well) into the upper chamber of the double-chamber tissue culture plates (pore size 0.4 μm, Transwell^®^; Corning, USA) and grown to confluency. 72 h later, the upper chamber of the Transwell^®^ was supplemented with a solution of FITC-conjugated dextran (FITC-dextran, Mr 40,000 Da, Sigma), at the final concentration of 10 μg/ml. After 60 min, a 20 μl aliquot was taken from the lower chamber, and intensity of the FITC fluorescence was measured using the Envision^®^ Multilabel Reader (Perkin Elmer) at 525 nm wavelength. Samples for all subsequent measurements were taken every 60 min. The experiment was performed in 4 parallels and repeated 6 times.

### Trypsinization experiments

To distinguish between intracellular and cell surface pools of VE-cadherin, HUVEC were rinsed in HBSS and incubated in 0.25 % trypsin/EDTA (GIBCO) at 37 °C to proteolytically remove cell surface VE-cadherin [14]. Trypsin was subsequently inactivated using EGM-2 media with serum, and cells were centrifuged at 2,000 rpm/4 °C. Cell pellets were dissolved in 2× Laemmli’s sample buffer for Western blotting analysis as described above. For controls, parallel cultures were harvested in 2× Laemmli’s sample buffer without trypsinization.

### Subcellular fractionation

Cellular fractions (membrane, cytosolic, and nuclear) were obtained using the Qproteome™ Cell Compartment kit according to manufacturer’s instructions. Cellular fractions were obtained with specific extraction buffer according to the manufacturer’s instructions (Qiagen). Protein concentration in each fraction was determined using the BCA protein assay kit (Pierce Biotechnology, Rockford, IL). Equal amounts of protein (20 μg) were subjected to 10 % SDS-PAGE and transferred to a polyvinylidene fluoride (PVDF) membrane. Purity and consistency of fractions was confirmed using antibodies against markers for different subcellular compartments: anti-EEA1 (to detect membrane and early endosomal proteins) and anti-LAMP1 (lysosomal marker to detect cytosolic proteins) (data presented in supplementary materials, Supplemental Fig. S1).

### Statistical processing of results

Statistical analysis was performed using Statistica 6.0 software. Values are expressed as mean ± standard error of mean (SEM) for normally distributed data and median and percentiles (25–75 %) for not normal data or small samples. If normality of data was confirmed (according to Kolmogorov–Smirnov test and Shapiro–Wilk’s W test) comparison of independent groups was performed by Student *t* test, if not or size of the analyzed sample was less than 10 cases—by Mann–Whitney U-criteria. Multiple comparisons were performed using one-way ANOVA for normally distributed data, otherwise—by Kruskall–Wallis test. Statistical significance was defined as *p*-value <0.05 and all reported statistical tests were two-tailed.

## Results

### Distribution of T-cadherin in HUVEC’s cell fractions after lentivirus transduction

**Fig. 1:**
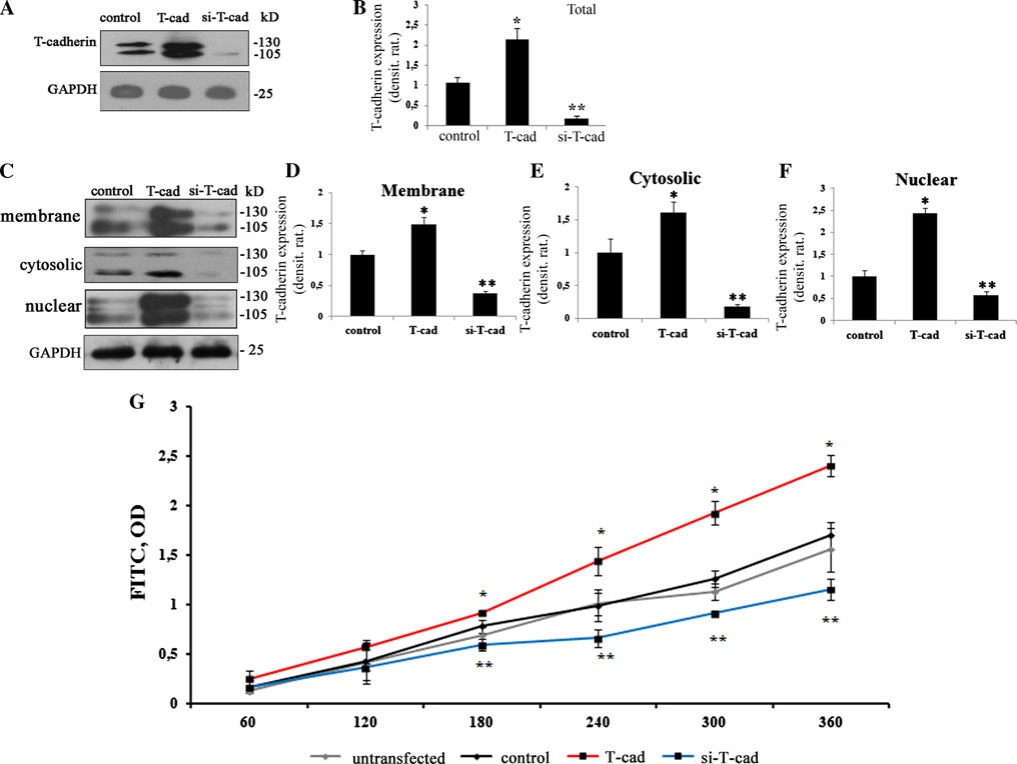
T-cadherin expression in HUVEC after lentivirus transduction. HUVEC cells at the 2nd passage were transduced by lentiviral construct for overexpression of T-cadherin (T-cad), T-cad silencing (si-T-cad) or control (control). Expression of T-cad was verified by Western blotting using anti-T-cad antibodies followed by densitometric analysis. **a** Expression T-cad in the total lysates of T-cad, si-T-cad, and control. In control T-cad was detected in precursor (130 kDa) and mature (105 kDa) forms. Upon overexpression, the content of precursor and mature forms of T-cad increased in T-cad cells compared to control; in si-T-cad, the expression of T-cad precursor was completely suppressed with only trace amounts of mature form detected. Western blotting of total lysates of three independent experiments was analyzed by densitometry analysis in (**b**) (*n* = 3; *, ** *P* at least <0.05). Protein loading was normalized using anti-GAPDH antibodies. **c** Cell lysates from HUVEC were fractionated and each cell fraction with equal protein loading was analyzed for T-cad content followed by densitometry (**d**), (**e**), (**f**). In control and in T-cad cells, T-cad was detected in membrane, cytosolic, and nuclear fractions in precursor and mature forms. However, T-cad expression was highly upregulated in T-cad cells compared to control; in si-T-cad expression of T-cad in all cellular fractions was significantly reduced. Western blotting was normalized by GAPDH level. Representative blots of three independent experiments are shown with densitometry analysis histograms (*n* = 3; *, ** *P* at least <0.05). **g** Endothelial monolayer permeability depends upon T-cad expression. Endothelial monolayer permeability was measured using FITC-dextran. FITC-dextran at the final concentration 10 μg/ml was applied to the upper chamber containing monolayers of untransfected cells (untransfected HUVEC), control, T-cad, or si-T-cad cells. Samples from the bottom chambers were probed every 60 min and tested at 525 nm wavelength. Data are presented as the mean ± SEM (* *P* < 0.001, *n* = 24). T-cad overexpression resulted in increased monolayer permeability and 6 h later it was 1.42-fold greater than in control. On the contrary, in si-T-cad cells permeability to FITC-dextran was markedly reduced, approximately by 1.47-fold compared to control

The role of T-cad in the regulation of endothelial barrier function was studied using lentivirus constructs for si-T-cad or enhancement of its native expression (T-cad). The content of T-cad in total lysates of HUVEC along with the membrane, cytosolic, and nuclear fractions was assessed after cell fractionation using Qproteome™ Cell Compartment kit and Western blotting (Fig. 1a, c) with densitometric analysis (Fig. 1b, d–f) 72 h after transduction. Purity and consistency of fractions was confirmed using antibodies against markers for different subcellular compartments: anti-EEA1 (to detect membrane and early endosomal proteins) and anti-LAMP1 (lysosomal marker to detect cytosolic proteins) (data presented in supplementary materials, Supplemental Fig. S1).

After HUVEC transduction with control virus, T-cad expression in the total lysates was observed in two forms: mature (mol wt 105 kDa) and precursor (mol wt 130 kDa) forms (Fig. 1a); in the membrane fraction T-cad was mainly present in the mature form; however, the precursor form was also detected; in the nuclear and cytosolic fractions both forms of T-cad were detected (Fig. 1c). Overexpression of T-cad resulted in a significant increase in T-cad content in total lysates (Fig. 1a), membrane, cytosolic, and nuclear fractions in both, the precursor and mature forms (Fig. 1c). The si-T-cad expression revealed virtually no precursor form of T-cad in the total lysates; however, the trace amounts of the mature form were still present (Fig. 1a). The absence of the precursor and the presence of the mature form of T-cad in total lysates indicate that after mRNA suppression the synthesis of new T-cad molecules has been practically stopped. After fractionation both forms of T-cad almost disappeared from the nuclear and cytosolic fractions, while in the membrane fraction mature form could be still detected (Fig. 1c). Thus, the overexpression of T-cad resulted in the overall increase in production of both, mature and precursor T-cad forms in all cell fractions, accounting for the T-cad synthesis and transport to the plasma membrane. Upon T-cad suppression firstly the precursor form disappeared, reflecting the suppression of mRNA with siRNA, while the trace amounts of mature T-cad still could be detected in the membrane fraction 72 h after transduction. These data confirmed that the created lentiviral constructs were effective and increased the overall expression of T-cad in T-cad cells; the construct for T-cad gene silencing significantly inhibited mRNA translation, while the lifetime of the T-cad on the membrane was longer than 72 h.

### T-cadherin expression regulates endothelial monolayer permeability

To study the role of T-cad in the regulation of endothelial barrier function, we investigated the influence of T-cad on monolayer integrity using endothelial permeability assay for FITC-dextran molecules. Upon transduction, HUVEC were cultured for at least 72 h until the formation of a long-term monolayer. The integrity of the monolayer was verified using light microscopy before each permeability experiment. FITC-dextran solution was added into the upper chamber of the Transwell system. Aliquots of the media from the lower chamber were taken and measured every 60 min (Fig. 1g). T-cad overexpression in HUVEC significantly elevates the monolayer permeability compared to the control so that 6 h later the difference increased by 1.42 times. In monolayer with si-T-cad the permeability to FITC-dextran was markedly reduced approximately by 1.47-fold compared to the control (Fig. 1g). Conclusively, these results suggest that T-cad expression regulates endothelial barrier function and its permeability to macromolecules: overexpression of T-cad increased endothelial permeability, while suppression of T-cad decreased it.

### T-cadherin induces gap formation and loss of VE-cadherin from cell–cell contacts in HUVEC

**Fig. 2:**
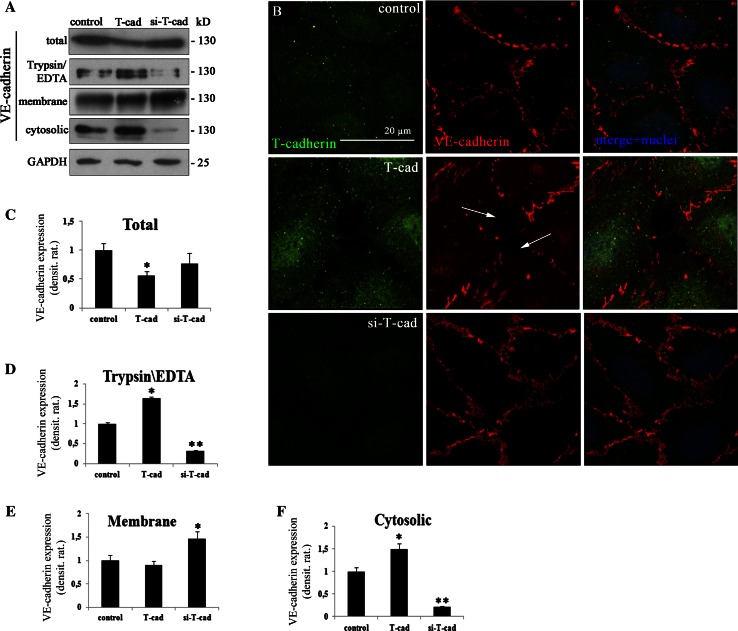
T-cad overexpression results in VE-cadherin accumulation in the cytosolic fraction of HUVEC cells and induces gap formation. **a** Expression of VE-cadherin in T-cad, si-T-cad, or control cells was analyzed by Western blotting with anti-VE-cadherin antibody and followed by densitometric analysis in (**c**), (**d**), (**e**), and (**f**). To distinguish between cell surface and intracellular pools of VE-cadherin, the membrane-bound VE-cadherin was proteolytically removed by incubation in trypsin/EDTA. Trypsinization revealed statistically significant increase in the content of intracellular VE-cadherin in T-cad cells compared to control, while in si-T-cad cells on the contrary the amount of intracellular VE-cadherin was reduced. Subcellular fractionation confirmed the reduction of VE-cadherin content in the membrane and its increase in the cytoplasm in T-cad cells. In si-T-cad cells VE-cadherin in the membrane fraction increased significantly, while in the cytosolic—it was almost undetectable. Western blotting data of VE-cadherin content in cell lysates was evaluated and normalized by GAPDH level. Representative blots of three independent experiments are shown and densitometry analysis histograms are given (*n* = 3; *, ** *P* at least <0.05). **b** To preserve the integrity of the cell membrane proteins, double-immunofluorescent staining of HUVEC using antibodies to T-cad and VE-cadherin was carried out without permeabilization. Representative examples of confocal image captures using the same confocal gain and offset settings are presented (T-cad—*green fluorescence*, VE-cadherin—*red fluorescence*). Nuclei appear *blue* after DAPI staining. VE-cadherin staining at cell–cell contacts in T-cad cells is much weaker than in control. Gaps at VE-cadherin cell–cell contacts are indicated by *white arrows*. *Bar* 20 μm

The regulation of VE-cadherin localization and integrity is essential for the endothelial barrier function [3]. We hypothesized that T-cad may regulate the endothelial permeability by changing the expression/localization of VE-cadherin in the cells. Expression of VE-cadherin in T-cad, si-T-cad, or control cells was assessed by Western blotting (Fig. 2a); and followed by densitometric analysis in total lysates, cell lysates after trypsinization and cells lysates after subcellular fractionation (Fig. 2c, d–f). Localization of VE-cadherin was evaluated by immunofluorescent staining with confocal microscopy (Fig. 2b). Western blotting of total lysates 72 h after cell HUVEC transduction showed statistically significant reduction in VE-cadherin content in T-cad cells compared to cont or si-T-cad cells (Fig. 2a, upper panel). Trypsinization experiments demonstrated that in contrast to cont or si-T-cad HUVEC, in T-cad HUVEC the main pool of VE-cadherin was located in the cytoplasm (Fig. 2a). This was further confirmed by subcellular fractionation and densitometric analysis of Western blotting results. In T-cad cells there was a small but statistically significant reduction in VE-cadherin content in the membrane fraction and obvious increase in the cytosolic fraction (Fig. 2d, e). In contrast, in si-T-cad cells the content of VE-cadherin significantly increased in the membrane fraction and decreased in the cytoplasm according to densitometry data (Fig. 2f). These results indicated that T-cad overexpression caused the reduction in the amount of VE-cadherin present on the cell membrane, while suppression of T-cad, on the contrary, resulted in the increased VE-cadherin localization on the membrane.

To visualize VE-cadherin localization in endothelial cells with different expression level of T-cad, we concentrated on VE-cadherin expression in the areas of intracellular contacts. Double-immunofluorescent staining with antibodies against T-cad and VE-cadherin was performed without permeabilization to preserve the integrity of cytoplasmic membranes. All images were captured using the same confocal gain and offset settings. In T-cad cells VE-cadherin staining at cell borders became intermittent (arrows in Fig. 2b); in addition VE-cadherin staining at the cell membrane was observed in the meshwork-like pattern. T-cad suppression, on the contrary, increased the thickness of VE-cadherin contacts, while in accordance with Western blotting and densitometry results almost no VE-cadherin could be detected in the cytoplasm (Fig. 2b). The disappearance of VE-cadherin from cell margins and disruption of the linear staining of VE-cadherin at intracellular junctions of T-cad cells was accompanied by the emerging gaps between the cells (arrows in Fig. 2b); while, in si-T-cad cells no gaps were observed.

According to Western blotting data T-cad expression exerted no obvious effect on N-cadherin or tight junction proteins occludin, claudin-5, or ZO-1 (Supplemental Fig. S2a, b).

Thus, our results suggest that T-cad overexpression in endothelial cells selectively disrupts VE-cadherin adhesive junctions and induce the formation of gaps between endothelial cells; this is accompanied by VE-cadherin accumulation in the cytoplasm and increased permeability of endothelial monolayer. Suppression of T-cad causes quite the opposite effect. We hypothesized that T-cad-mediated disruption of VE-cadherin adhesive contacts could result from the increased VE-cadherin endocytosis.

### T-cadherin overexpression induces VE-cadherin internalization via clathrin-dependent pathway and lysosomal degradation

**Fig. 3:**
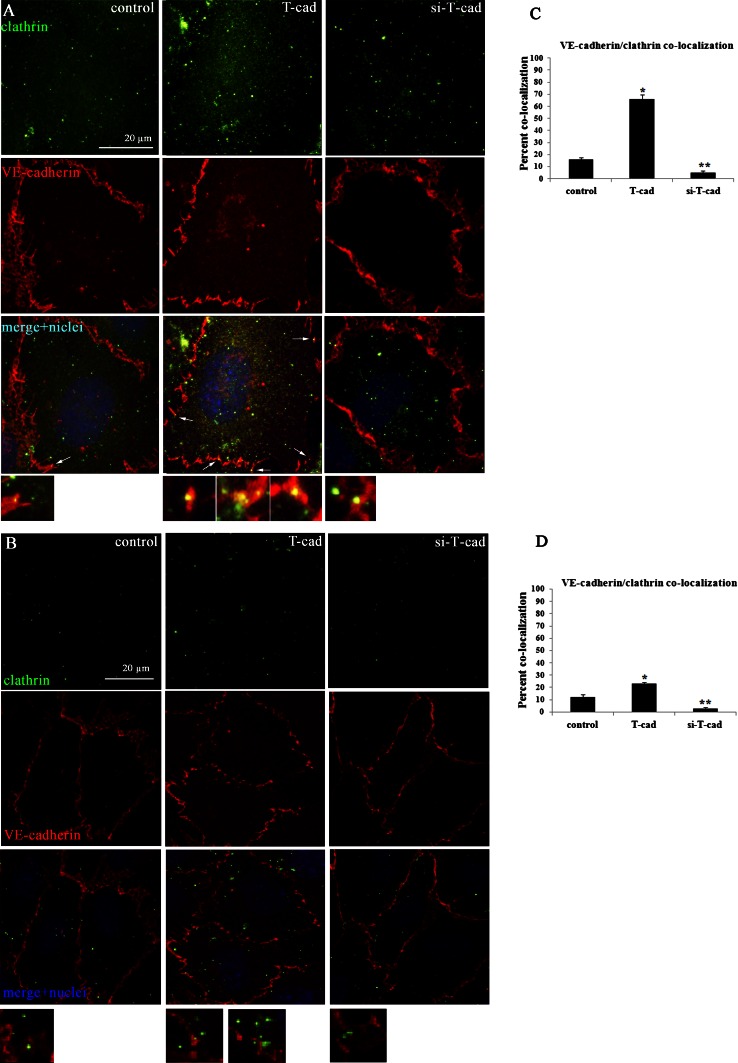
T-cad overexpression induces VE-cadherin internalization via clathrin-dependent pathway in HUVEC ells. **a** Confocal high-resolution images of T-cad, si-T-cad, or control HUVEC double-immunostained with antibodies against clathrin (*green fluorescence*) and VE-cadherin (*red fluorescence*). Nuclei appear blue after DAPI staining. *Yellow fluorescence* corresponds to VE-cadherin and clathrin co-localization (*white arrows*, also in the lower panel). **b** To inhibit clathrin-dependent endocytosis, control, T-cad, and si-T-cad cells were treated with 80 μM dynasore for 2 h prior to staining. Cells were double-stained with anti-clathrin (*green fluorescence*) and anti-VE-cadherin (*red fluorescence*) antibodies. Nuclei were counterstained with DAPI. Lower panels demonstrate the absence of clathrin and VE-cadherin co-localization in the cytoplasm of endothelial cells. Images were captured using high-resolution confocal microscope with equal gain and offset settings. *Bar* 20 μm. **c** and **d** Quantification of co-localization of VE-cadherin with clathrin. Co-localization was acquired by immunofluorescence confocal analysis of representative images of four independent experiments in three triplicates and quantified using the ImageJ co-localization plug-in. Values are displayed as mean ± SEM (*n* = 12; *, ** *P* at least <0.05)

To test the hypothesis that T-cad overexpression induces clathrin-dependent endocytosis of VE-cadherin, we tested the co-localization of VE-cadherin with clathrin using double-immunofluorescent staining of HUVEC. Confocal high-resolution images were captured with equal gain and offset settings. As shown in Fig. 3a, in T-cad cells VE-cadherin co-localized extensively with clathrin on the surface of endothelial cells (clathrin-coated pits are shown by white arrows and depicted in the lower panel of Fig. 3a) as well as in the cytoplasm; while, in control and si-T-cad cells co-localization of VE-cadherin with clathrin was less pronounced. This co-localization effect appeared to be specific for clathrin and VE-cadherin, since no co-localization of VE-cadherin with caveolin-1 could be detected in T-cad, control, or si-T-cad cells (Supplemental Fig. S2c).

To confirm this, we applied inhibitory analysis using dynasore at the final concentration 80 μM [15]. Experiments with dynasore showed the decreased clathrin staining at the complete absence of VE-cadherin co-localization with clathrin in the membrane fraction (Fig. 3b, lower panel) and in the cytoplasm of T-cad, si-T-cad, and control cells (Fig. 3b). This data confirmed our hypothesis that overexpression of T-cad leads to VE-cadherin endocytosis via clathrin-dependent pathway.

**Fig. 4:**
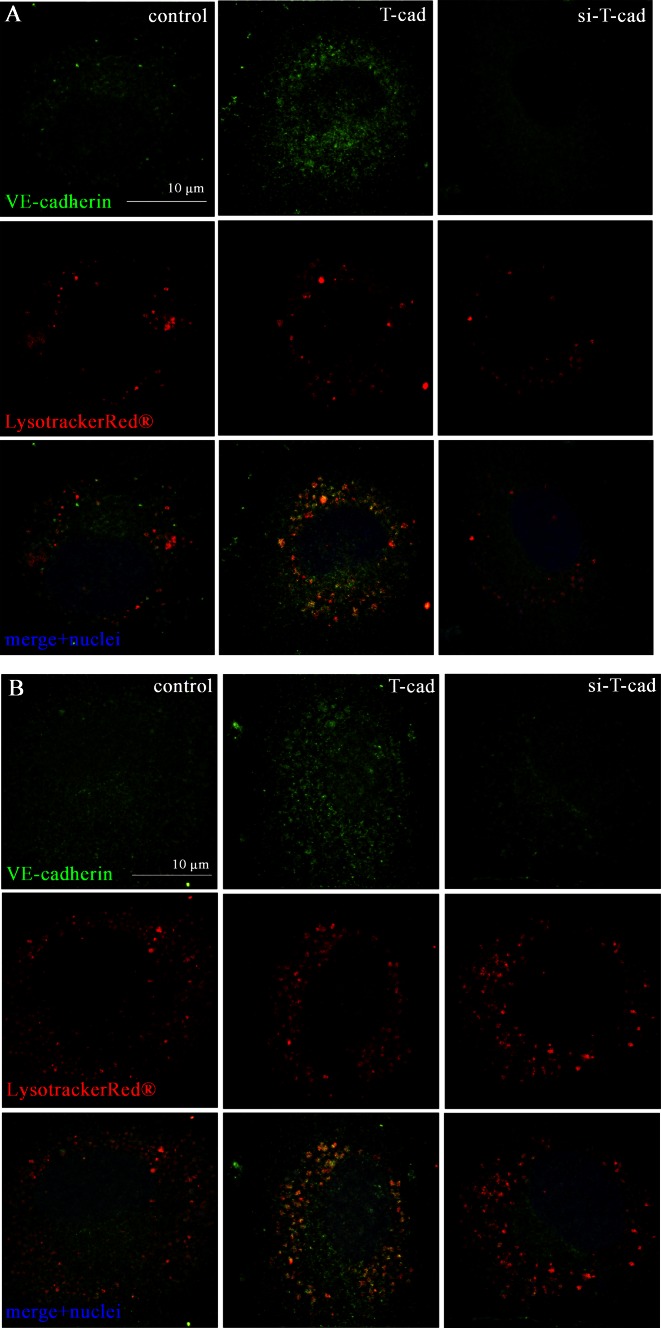
VE-cadherin localizes in lysosomes in T-cad overexpressing endothelial cells. Intracellular localization of VE-cadherin and its co-localization with lysosomes was revealed by immunofluorescence and confocal high-resolution analysis in equal gain and offset settings. Optical sections in (**a**) and (**b**) have been acquired across the nucleus. Control, T-cad, and si-T-cad endothelial cells were preincubated with LysotrackerRed^®^ (*red fluorescence*) for 60 min in (**a**) and 120 min in (**b**), and then immunostained using antibodies against VE-cadherin (*green fluorescence*). Nuclei appear blue after DAPI staining. Co-localization of VE-cadherin and lysosomes marker corresponds to *yellow fluorescence*. Noteworthy, co-localization of VE-cadherin and lysosome marker is observed in most cases in T-cad cells, while in control and especially in si-T-cad it is not observed. *Bar* 10 μm

To determine whether the internalized VE-cadherin was subjected to further degradation in lysosomes we carried out co-localization experiments of VE-cadherin and 75 nM LysotrackerRed^®^, a fluorescent lysosome marker, as described before [16]. Control, T-cad, and si-T-cad HUVEC were preincubated with LysotrackerRed^®^ for 60 min (Fig. 4a) and 120 min (Fig. 4b), immunostained using antibodies against VE-cadherin and analyzed using high resolution confocal microscopy analysis. After pre-incubation with lysosome marker (especially after 120 min), co-localization of VE-cadherin with LysotrackerRed^®^ was much more pronounced in the cytoplasm of T-cad cells than in control and especially in si-T-cad cells (Fig. 4b), thus pointing to enhanced internalization and degradation of VE-cadherin in T-cad cells [14, 17].

Taken together these data indicate that T-cad overexpression results in the increase in clathrin-dependent endocytosis of VE-cadherin followed by its subsequent degradation in lysosomes.

### T-cadherin regulates phosphorylation level of VE-cadherin (Y731) in HUVEC

Intracellular domain of VE-cadherin is known to become phosphorylated on tyrosine residues [18, 19]. We assumed that overexpression of T-cad can lead to tyrosine phosphorylation of VE-cadherin resulting in its subsequent degradation in lysosomes. VE-cadherin domain containing Y731 was predicted to interact with β-catenin, while Y658—to mediate VE-cadherin binding to p120^ctn^ [20–22]. Therefore, the question was whether the phosphorylation of these two residues correlates with T-cad-dependent VE-cadherin internalization.

**Fig. 5:**
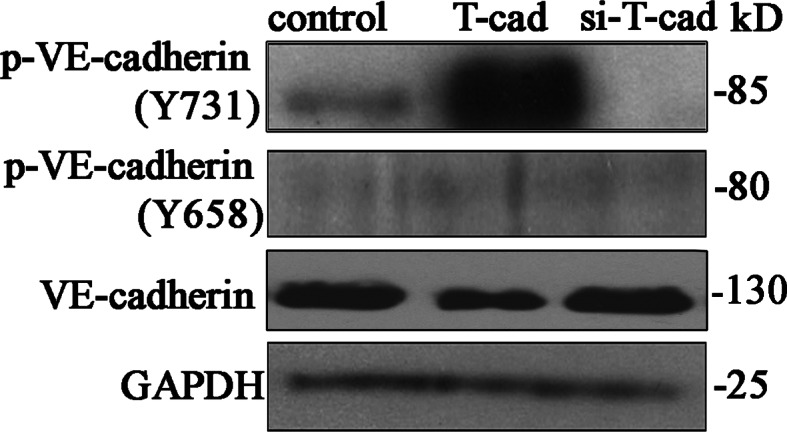
VE-cadherin becomes phosphorylated on Tyr731 but not on Tyr658 upon T-cad overexpression. The amount of phospho-VE-cadherin (p-VE-cadherin) was analyzed in T-cad, si-T-cad, and control cells using Western blotting. Phosphorylation of VE-cadherin was evaluated in total lysates using antibodies against p-VE-cadherin on Y731 and Y658. Western blotting data of phospho-VE-cadherin content in cell lysates was evaluated by total VE-cadherin and GAPDH level. Representative blots of three independent experiments are shown

To answer this, total lysates of cont, T-cad, and si-T-cad HUVEC were analyzed by Western blotting using phospho-specific antibodies directed against cytoplasmic domain of VE-cadherin phosphorylated on either Y658 or Y731. Antibodies to phospho-VE-cadherin have been previously used to reveal the phosphorylation of VE-cadherin on Y658 or Y731 [23, 24]. It was found that T-cad overexpression resulted in dramatic increase in phosphorylation level of VE-cadherin on Y731 compared to control, while phosphorylation on Y658 remained unchanged (Fig. 5). In si-T-cad cells virtually no Y731-phosphorylated form of VE-cadherin was present, while Y658 phosphorylation status was unchanged. These data indicate that T-cad overexpression resulted in enhanced phosphorylation of VE-cadherin on Y731, but not on Y658.

**Fig. 6:**
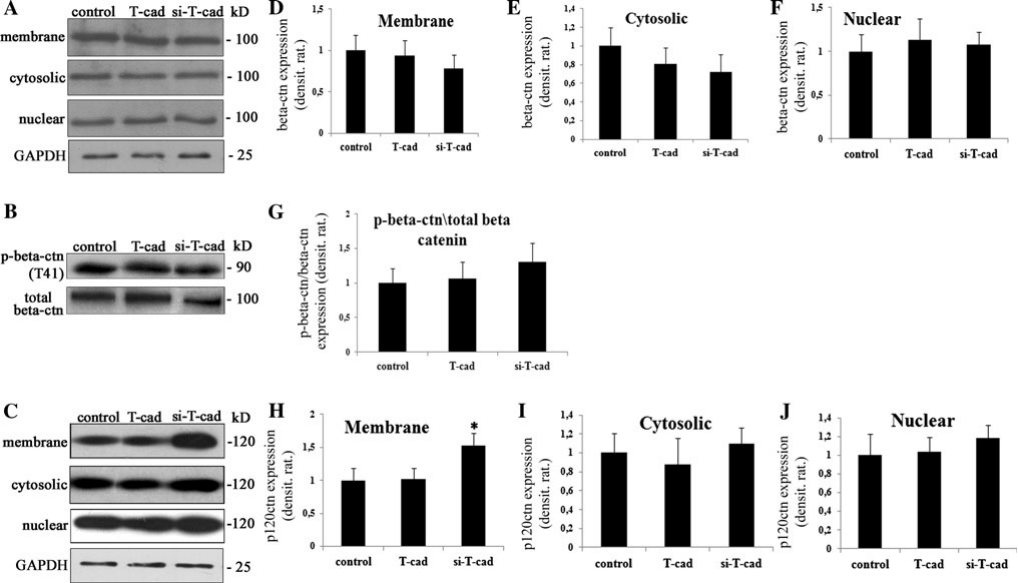
Overexpression of T-cad in endothelial cells had no effects on β-catenin, while T-cad silencing resulted in the increase of p120^ctn^ content in the cell membrane. Representative blots of three independent experiments are shown and densitometry analysis histograms are given (*n* = 3; *P* > 0.05). **a** Expression of β-catenin after subcellular fractionation was analyzed by Western blotting and densitometry analysis in (**d**), (**e**), and (**f**). **b** β-catenin phosphorylation level were determined in total lysates of control, T-cad, and si-T-cad cells using antibodies against p-β-catenin on T41 (p-beta-ctn T41) using Western blotting and densitometry analysis in **g**. T-cad expression exerts no effect on β-catenin localization in cellular compartments or β-catenin phosphorylation. Data was normalized by total β-catenin (beta-ctn) content. **c** The content of p120^ctn^ in subcellular fractions was analyzed by Western blotting and densitometry analysis in (**h**), (**i**), and (**j**). Suppression of T-cad resulted in the increase of p120^ctn^ in the membrane fraction compared to control and T-cad. The level of p120^ctn^ in cytosolic and nuclear fractions in T-cad cells was similar to control

We tested if T-cad expression impacts localization and phosphorylation status of β -catenin and p120^ctn^ (Fig. 6). For that cell fractionation and Western blotting with densitometric analysis was performed that revealed no statistically significant difference in β-catenin content in the nuclear and cytosolic fractions upon T-cad overexpression compared to control (Fig. 6a, d–f), though there was a tendency in the increase in β-catenin content in the nuclear fraction (Fig. 6f). The amount of membrane and phosphorylated β-catenin was also similar between control and T-cad cells (Fig. 6b, g). No statistically significant difference was found in the content of β-catenin in the nuclear, cytosolic, or membrane fractions of si-T-cad and control cells (Fig. 6a, d–f). Also, there was no statistically relevant difference in the amount of phosphorylated β-catenin between si-T-cad and control cells (Fig. 6b, g).

Interestingly, while the amount of p120^ctn^ was the same in the cytosolic, membrane, and nuclear fractions of control and T-cad cells, the content of p120^ctn^ in the membrane fraction of si-T-cad was dramatically increased (Fig. 6c, h, j, k). These suggest the possible involvement of p120^ctn^ in the enhanced VE-cadherin-mediated barrier function as judged by the decreased endothelial cell permeability to FITC-dextran in cells with downregulated T-cad. Presumably, p120^ctn^ accumulation in the membrane area of si-T-cad cells could prevent the internalization of VE-cadherin and stabilize the adhesive contacts. By and large these results indicate that T-cad overexpression induces Y731 phosphorylation of VE-cadherin, which may be the mechanism underlying the increased clathrin dependent internalization of VE-cadherin and increased endothelial monolayer permeability. To determine the exact role of β-catenin and p120^ctn^ in this process, further research is needed.

### T-cadherin activates small G proteins RhoA, Rac1, Cdc42, and induces actin stress fibers formation in HUVEC

**Fig. 7:**
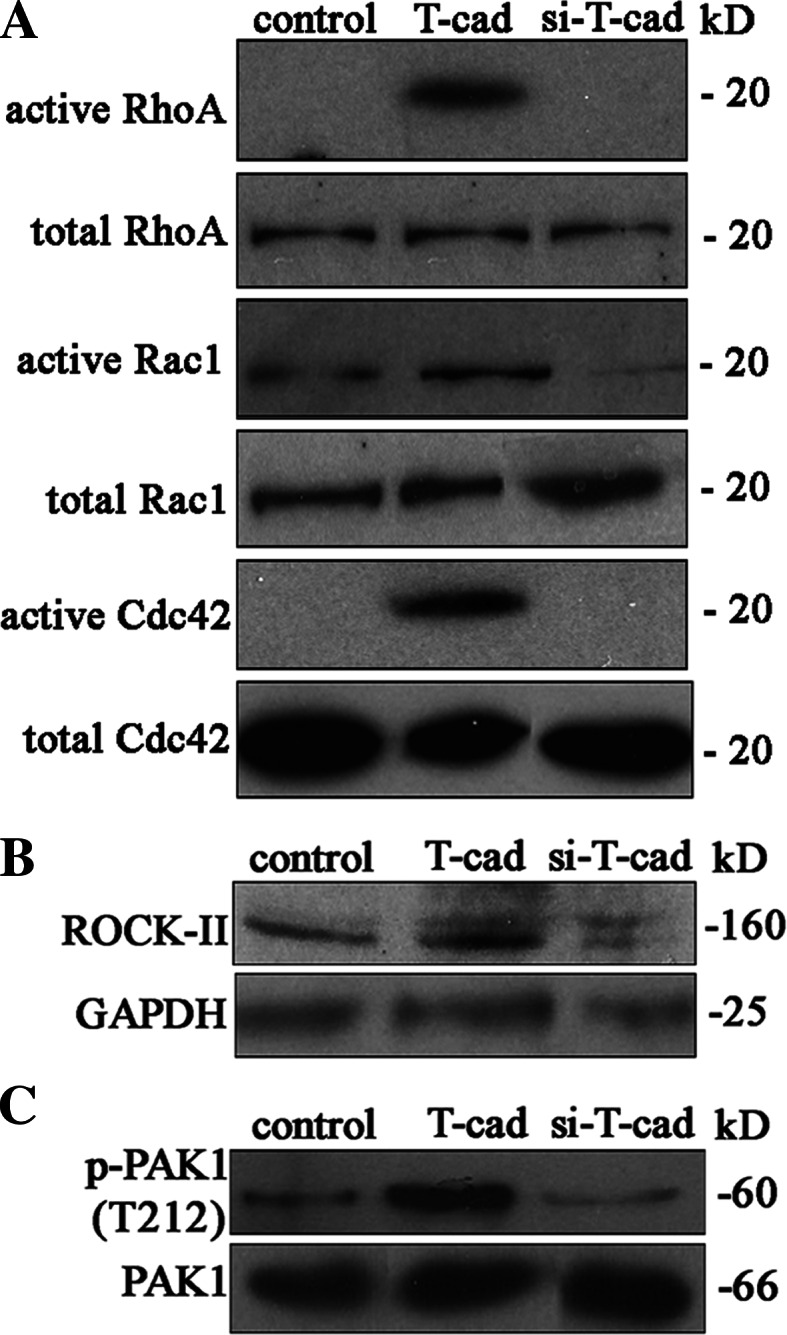
T-cad activates small G proteins and their downstream signaling adaptors ROCK-II and PAK1 in HUVEC. **a** Active forms of Rho GTPases were isolated from the whole lysates of T-cad, si-T-cad, or control cells by precipitation with Sepharose. T-cad overexpression resulted in the robust activation of RhoA, Rac1, and Cdc42 compared to control or si-T-cad cells. The total protein content of RhoA, Rac1, and Cdc42 remained unchanged. The level of activation of Rho GTPases downstream targets was evaluated by the content of active ROCK-II (**b**) and PAK1 (**c**) in total lysates of T-cad, si-T-cad, or control cells. T-cad overexpression resulted in the increase of ROCK-II content and the amount of PAK phosphorylated on threonine 212 (T212) as compared to control cells. In si-T-cad the content of ROCK-II and the level of PAK1phosphorylation were reduced compared to control cells

Activation of Rho GTPases (RhoA, Rac1, and Cdc42) is known to influence endothelial barrier permeability [25, 26]. In order to identify the possible involvement of Rho GTPases in the T-cad-mediated effects on endothelial permeability we isolated active forms of Rho GTPase by precipitation using lysates of control, T-cad, and si-T-cad HUVEC. It was found that T-cad overexpression in HUVEC resulted in the activation of RhoA, Rac1, and Cdc42; while, the total protein content of Rac1, Cdc42, and RhoA remained unchanged (Fig. 7a). Noteworthy, no active forms of RhoA and Cdc42 were detected in control or si-T-cad cells (Fig. 7a).

Rho family associated kinases, ROCK-II and PAK1 are direct downstream targets of Rho family GTPases (ROCK-II is activated by RhoA, while PAK1 is a target kinase of activated Rac1 and Cdc42) [27]. T-cad overexpression correlated nicely with an increase in the ROCK-II content and the level of phosphorylated PAK1 comparing to control (Fig. 7b). In si-T-cad HUVEC the content of ROCK-II and the degree of phosphorylation of PAK1 were reduced compared to control cells (Fig. 7c).

The data on Rho GTPases, ROCK-II and PAK1 activation was further confirmed by functional rearrangement of the cytoskeleton HUVEC. As presented in supplementary materials T-cad overexpression induced stress fiber formation, while in si-T-cad cells actin stress fiber formation was less pronounced compared to control (Supplemental Fig. S3a). We suggested that ROCK-II could be required for the stress fiber formation induced by T-cad overexpression. This hypothesis was further challenged using Y27632—inhibitor of Rho kinase ROCK. Y27632 induced relaxation of stress fibers in T-cad HUVEC (compare Supplemental Fig. S3a and Supplemental Fig. S3b), suggesting that ROCK-II acts downstream of RhoA and mediates T-cad effects on the actin cytoskeleton.

Also our data on the increased content of ROCK-II in the nuclei of T-cad cells (Supplemental Fig. S3a) indicate to the potential involvement of ROCK-II activation and nuclear signaling in these cells, although more studies are needed to establish its nuclear-specific function.

We also addressed the question if the observed effects of T-cad on Rho GTPase activation and cytoskeletal reorganization were specific. For that we analyzed the phosphorylation level of Src, p38, and ERK1/2. Western blotting revealed that overexpression of T-cad as well as downregulation of T-cad had no significant effect on Src, p38, and ERK1/2 activation, since there was no pronounced change in the contents of p-Src, p-p38, and p-ERK1/2 at the constant total level of these proteins (Supplemental Fig. S4). The presented data indicate that T-cad overexpression in HUVEC reduces endothelial barrier function suggesting the involvement of VE-cadherin clathrin-dependent endocytosis and degradation in lysosomes, activation of Rho GTPases and their downstream proteins followed by enhanced actin stress fiber polymerization.

## Discussion

The accumulated evidence indicated that there is a correlation between upregulated T-cad expression in vascular cells and the progression of cardiovascular disease such as atherosclerotic lesions and restenosis [11, 12, 28]. In addition, the elevated expression of T-cad was suggested to be involved in the pathogenesis of insulin resistance in endothelial cells through the mechanism of chronic activation of the Akt/mTOR-dependent negative feedback loop and the increased degradation of insulin receptor substrate [29]. These data suggest that T-cad upregulation may precede or reflect the activation/dysfunction of endothelial cells, enhanced permeability of endothelium and vascular injury in the above-mentioned pathological conditions.

We describe here a new mechanism of regulation of endothelial permeability involving T-cad. The elevated expression of T-cad impairs the endothelial barrier function via the activation of Rho GTPases signaling, VE-cadherin phosphorylation with subsequent clathrin-dependent endocytosis and degradation in lysosomes, and reorganization of the actin cytoskeleton. Suppression of T-cad causes the opposite effect. These data are in accordance with the previously published results. Complex signaling network that involves T-cad in the regulation of endothelial barrier was suggested by Andreeva and co-authors. They showed that T-cad is involved in the reduction of the transendothelial resistance upon stimulation with thrombin or serum; however, the detailed mechanisms remained unknown [30]. Earlier data obtained in our lab also indicated that the suppression of T-cad resulted in the decreased permeability of the endothelial monolayer to macromolecules in vitro [7]. Previously, using a semiquantitative evaluation of the fluorescence upon staining with antibodies to VE-cadherin we revealed a tendency for increase in VE-cadherin content in the zone of adhesive contacts in si-T-cad cells [7]. In the present paper we used both, HUVEC overexpressing T-cad and HUVEC with suppressed T-cad expression and applied a combination of methods including cell fractionation, Western blotting with densitometric analysis and confocal microscopy to reveal the details of the mechanisms of T-cad-mediated regulation of endothelial permeability.

Our results on the presence of T-cad in the nuclear fraction correlate with confocal microscopy and nuclear fractionation data previously published by Andreeva and co-authors [30], who showed that T-cad is normally detected in the nucleus of endothelial cells and HEK 293. Primary structure analysis of T-cad revealed that it is enriched in basic residues and is predicted to have nuclear localization [30]. These points to the fact that T-cad localization in the nuclear fraction is not an off-target effect of cell transduction; however, the physiological significance of this phenomenon is unclear. The T-cad location in the nucleus supports the notion that T-cad could be involved in the modulation of gene transcription as it has been shown before for E-cadherin [31] and urokinase receptor uPAR- another GPI-anchored protein [32]. The data on upregulation of cyclin D1 in endothelial cells [33] and activation of serum-response elements in NIH3T3 fibroblasts upon T-cad overexpression [7] are in favor of the assumption that T-cad is transported into the nucleus to regulate gene expression.

In case of vascular damage under pathological conditions, such as atherosclerosis, hypertension, and diabetes mellitus, the endothelial layer could be seriously impaired, endothelial cells retracted and endothelial barrier function compromised followed by the obvious disruption of blood vessels. However, many agents such as histamine, thrombin, cytokines, antioxidants, VEGF, and activated neutrophils reversibly increase endothelial permeability [5].

Thus, it has been demonstrated that VEGF interaction with VEGFR2, results in the activation of small GTPase Rac through Src-dependent pathway. Rac activity causes the direct activation of its downstream target p21-activated kinase PAK, which, in turn, promotes phosphorylation of VE-cadherin on serine S665 [34] or tyrosine Y685 [35, 36] and acto-myosin contraction. In another paper, it was shown that the engagement of antigen-activated lymphocytes induced phosphorylation of VE-cadherin on Y645, Y731, and Y733 followed by actin reorganization, and that this phosphorylation was also mediated by Rho GTPase activation [21]. Here, we revealed that T-cad overexpression resulted in strong phopshorylation of VE-cadherin precisely on Y731 (but not on Y658) (Fig. 5) followed by increased VE-cadherin endocytosis and endothelial permeability. Several studies indicated that VE-cadherin is preferentially internalized via a clathrin-dependent pathway [14, 17, 34, 37]. It was indeed the clathrin-dependent endocytosis of VE-cadherin that was associated with T-cad expression as judged by co-localization of VE-cadherin with clathrin (but not caveolin-1). This was confirmed by inhibitory analysis with dynasore (Fig. 3). Dynasore is a cell-permeable inhibitor of dynamin which blocks clathrin-dependent endocytosis [15]. Further experiments showed co-localization of VE-cadherin with lysosome marker. Localization of VE-cadherin in the lysosomal compartment indicates that VE-cadherin undergoes degradation in lysosomes and is not subjected to recycling [14, 17].

Though in the majority of papers VE-cadherin internalization was found to proceed via clathrin-dependent pathway as the primary way of endocytosis for classical cadherins [34, 37], there is evidence that caveolin-1 can also contribute to the thrombin-induced decrease in the barrier function [38]. We found no co-localization of caveolin-1 and VE-cadherin (Supplemental Fig. S2c) in control, T-cad, or si-T-cad cells, indicating that caveolin-1 is not involved in T-cad-mediated regulation of cell permeability.

In endothelial cells, cytoplasmic domain of VE-cadherin binds β-catenin, p120^ctn^, α-catenin; both β-catenin and α-catenin connect the cadherin complex directly to cytoskeleton mediating strong intracellular adhesion [3, 5]. Previously, VE-cadherin domain containing Y731 was predicted to interact with β-catenin, while Y658—with p120^ctn^ [20–22]. Phosphorylation of VE-cadherin on these sites disrupts the binding of β-catenin and p120^ctn^. Consequently, β-catenin and p120^ctn^ which are released from the cadherin complex may be phosphorylated and degraded in proteosomes of the cytoplasm; otherwise, they can be transported to the nucleus where they participate in regulation of gene expression [39, 40]. Our previously obtained results using a semiquantities evaluation of the fluorescence intensities revealed the difference in the staining against β-catenin in the nuclei and cytoplasm in si-T-cad and control cells [7]. Using a more precise cell fractionation and Western blotting with densitometric analysis we detected no statistically significant difference in β-catenin content between the nuclear, membrane, and cytosolic fractions, though there was a tendency in β-catenin increase in the nuclear fraction of T-cad cells compared to control. The amount of phosphorylated β-catenin was also similar between control and T-cad cells (Fig. 6b); thus, supporting the assumption that the released from VE-cadherin complex β-catenin is not subjected to degradation in proteosomes. These data are in accordance with published data on T-cad overexpression resulting in activation and stabilization of β-catenin, which is not subjected to proteosome degradation and is translocated into the nucleus to regulate gene expression [33]. However, the physiological role of β-catenin in T-cad-mediated effects is still unclear and needs further studies. In the earlier papers, it was shown that p120^ctn^ protects VE-cadherin form degradation thus enhancing the stability of the cadherin-cytoskeleton complex [14, 17, 41]. Interestingly, while T-cad overexpression had no effect on p120^ctn^ expression/localization, in si-T-cad cells we detected the increase in p120^ctn^ content in the membrane fraction, suggesting that in the absence of T-cad, p120^ctn^ could become associated with VE-cadherin and enhance endothelial barrier function (Fig. 6c). These data correlates with recently published results by Kowalczyk and co-authors that binding of p120^ctn^ to VE-cadherin inhibits endocytosis of VE-cadherin [42].

Rho GTPases (RhoA, Rac1, and Cdc42) have received much attention as key regulators of endothelial barrier function via controlling the phosphorylation status of cadherin–catenin complex, stress fiber formation, and regulation of acto-myosin complex activity [25, 26]. RhoA itself induces the formation of actin stress fibers as well as activates Rho kinases resulting in phosphorylation of the myosin light chain and cell contraction [25, 43]. Cdc42 and Rac1bind to and activate PAK1, which controls contractility through its effects on LIM kinase [43, 44]. Earlier we have demonstrated that in NIH3T3 fibroblasts T-cad expression induced Rac1 and Cdc42 signaling, but not RhoA [7]. However, it is well known that the exact activation pattern of Rho GTPases and the consequences of GTPase signaling depend profoundly on cellular context [45]. Therefore, in the present study we used HUVEC and assessed the activation status of Rho GTPases in control, T-cad, and si-T-cad cells. T-cad overexpression resulted in the activation of RhoA, Rac1, and Cdc42, whereas no activation was found in control or si-T-cad cells. Using Western blotting and inhibition analysis we demonstrate that T-cad overexpression results in activation of direct downstream targets of Rho GTPases, PAK1, and ROCK (Fig. 7). Subsequent confocal microscopy and inhibitory analysis confirmed that activation of RhoA, Rac1, and Cdc42 signaling pathways resulted in activation of their downstream adaptor proteins resulted in the actin stress fiber assembly (Supplemental Fig. S3).

In addition to the regulation of the cytoskeleton, Rac1/Cdc42 signaling pathway is known to be involved in the activation of p38/MAPK and ERK1/2 that regulate the activity of genes responsible for cell motility, apoptosis, inflammation as well as cell proliferation and differentiation [46, 47]. The results of our experiments indicate that the change in T-cad expression doesn’t affect the phosphorylation of p38 or ERK1/2 in HUVEC. Previously, Src activation was shown to induce phosphorylation of VE-cadherin on Y658, Y685 [5, 35], and Y731 [20] resulting in uncoupling of the VE-cadherin from catenins and cytoskeleton. However, we detected no activation/phosphorylation of Src in HUVEC upon expression in T-cad or si-T-cad cells (Supplemental Fig. S4). These results suggest that in endothelial cells T-cad mediates Rho GTPase-dependent signaling effects, causing changes in the organization of the cytoskeleton, but has no effect on the activity of p38, Erk1/2, or Src.

Thus, the above data indicate that in HUVEC T-cad overexpression diminishes endothelial barrier function through VE-cadherin clathrin-dependent endocytosis and degradation in lysosomes, activation of Rho GTPases and enhanced actin stress fiber polymerization.

**Fig. 8:**
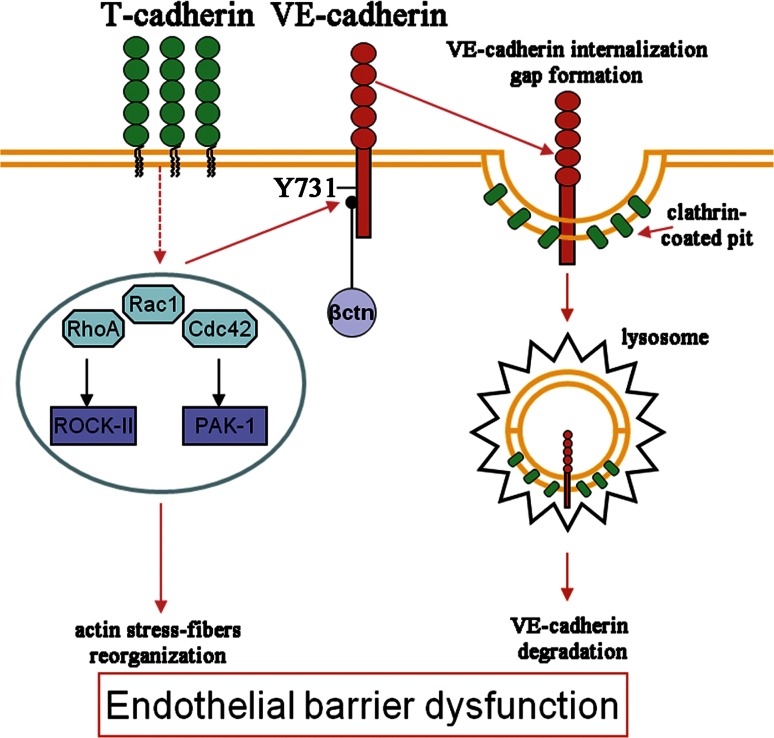
Final scheme. See explanation in text

The present study offers a new insight on T-cad as a regulator of endothelial permeability through the mechanism summarized in Fig. 8. Pathological processes such as inflammation, atherosclerosis, hypertension, and diabetes mellitus cause a dissociation and rearrangement of endothelial cells junctions and results in enhancement of endothelial permeability. This study for the first time directly demonstrates negative effects of the elevated T-cad expression on the endothelial barrier function and positive effects of T-cad suppression manifested as the reduction in endothelial permeability. Our results suggest the feasible link between elevated level of T-cad in endothelial cells of blood vessels and progression of cardiovascular disease characterized by the impaired barrier function.

## Electronic supplementary material

Below is the link to the electronic supplementary material.

Supplementary material 1 (DOC 38 kb)

Supplementary material 2 (TIFF 98103 kb)

Supplementary material 3 (TIFF 98104 kb)

Supplementary material 4 (TIFF 98103 kb)

Supplementary material 5 (TIFF 133536 kb)

## Abbreviations

GPI: Glycosilphosphoinositol
T-cad: T-cadherin
si-T-cad: Suppression of native T-cadherin
