# Supplementary material for: Novel mechanism regulating endothelial permeability via T-cadherin-dependent VE-cadherin phosphorylation and clathrin-mediated endocytosis
Source: Mol Cell Biochem. 2013 Oct 18;387(1):39–53. doi: 10.1007/s11010-013-1867-4 (PMC3904039; doi:10.1007/s11010-013-1867-4)
Supplement: Supplementary file 1 — Supplementary material 1 (DOC 38 kb) [file 11010_2013_1867_MOESM1_ESM.doc]

**SUPPLEMENTAL MATERIAL**

**Novel mechanism regulating endothelial permeability via T-cadherin dependent VE-cadherin phosphorylation and clathrin mediated endocytosis.**

Ekaterina V. Semina^a^, Kseniya A. Rubina^b^, Veronika Yu. Sysoeva^b^, Pavel N. Rutkevich^a^, Natalia M. Kashirina^a^, Vsevolod A. Tkachuk^b^.

^a^Russian Cardiology Research-Industrial Complex, Ministry of Health, 3rd Cherepkovskaya st. 15a, 121552 Moscow, Russia; Tel +7(495) 414-6713, Fax: +7(495) 414-6712.

^b^Faculty of Basic Medicine, Lomonosov Moscow State University, Lomonosov ave 31/5, 119192 Moscow, Russia; Tel/fax: +7(495) 932-9904.

**MATERIALS AND METHODS**

**Generation of lentiviral constructs**

The lentiviral T-cadherin expressing construct pSIH-H1-Tcad was prepared using a pSIH1-H1-puro lentiviral vector (System Bioscience, USA) as follows. The puromycin resistance gene was excised, and cloned human T-cadherin cDNA (GenBank Accession No. NM_001257.3) was placed under the control of cytomegalovirus immediate early promoter P_CMV_. The empty vector pSIH-H1-control with no insertion was used for control transduction. For RNA interference mediated T-cadherin gene silencing the lentiviral construct pSIH-H1-puro-siTcad was used. This construct was prepared to express short hairpin RNA (shRNA) directed to the T-cadherin target sequence GGTGAGTGTCTTAGCATAT, which is located at the 3’-untranslated region of the human T-cadherin mRNA. The shRNA expression is driven by the promoter Р_Н1_ of human RNAse H1 RNA component.

To produce VSVG-pseudotyped lentiviral particles, 15x10^6^ НЕК293-TN cells (System Biosciences) were transfected with 6 µg of the lentiviral vector DNA and 30 µg of the packaging plasmid mixture pPACK (System Biosciences) using Lipofectamine 2000 (Invitrogen). 72 H after transfection, the culture medium was collected and pseudoviral particles were pelleted by centrifugation for 5 h at 60000 g, +4^0^C. The precipitate was then resuspended in a small volume of PBS, aliquoted and stored at –80^0^C until use. Each lot of the pseudoviral particles was characterized by transducing the model H1299 human lung carcinoma cells and estimating the resultant multiplicity of infection (MOI, see below). Only the viral lots which gave MOI higher than 0.5 were chosen for further experiments. For lentiviral transduction the cells growing in 6-well plate (premature monolayer) were incubated for 24 h with the 300 µl aliquote of the pseudoviral particles diluted in 2 ml of fresh culture medium in the presence of 5 µg/ml polybrene (Sigma-Aldrich). After that, the medium was changed, and the cells were cultured for 2 weeks before testing.

To estimate the multiplicity of infection of the transduced cells, genomic DNA was isolated from these cells and analyzed by PCR with GGGGACTGGAAGGGCTAATTC and TGCGTCGAGAGAGCTCTGGTT primers. These primers were designed to recognize only the integrated copies of the lentiviral construct. The reaction was carried out for 31 cycles under the following conditions: template denaturation (30 sec at 94^0^C), primer annealing (60 sec at 60^0^C), and elongation (45 sec at 72^0^C), and the amplification products were separated in 1.5% agarose gel in the presence of 1 μg/ml ethidium bromide. The number of lentiviral vector copies integrated into the genome (equivalent to MOI) was estimated by the fluorescence of the amplified DNA fragment. As calibration standard, we used genomic DNA isolated from transduced H1299 human lung carcinoma cell line bearing exactly one integrated copy of lentiviral vector. To verify the equal quantities of genomic DNA analyzed, another PCR was performed using ACCACAGTCCATGCCATCAC and TCCACCACCCTGTTGCTGTA primers specific for human glyceraldehyde-3-phosphate dehydrogenase (GAPDH) gene. The reaction was carried out for 25 cycles under the same conditions.

**RESULTS**

**T-cadherin overexpression induces actin stress fibers formation**

Actin stress fibers and ROCK-II expression were visualized by confocal microscopy in control, T-cad and si-T-cad HUVEC. In control cells actin was present in the form of actin stress fibers, mostly distributed in the zig-zag pattern along the long axis of a cell or ending in the middle of a cell. T-cadherin overexpression induced stress fiber formation and modified their shape and distribution within the cells, inducing deposits of thick actin bundles along the cell axis as indicated by the increased phalloidin staining (green fluorescence). T-cadherin overexpression also resulted in the pronounced ROCK-II staining compared to control (red fluorescence) (Supplemental Fig. S3a and S3b). In contrast, in si-T-cad HUVEC ROCK-II expression and actin stress fiber formation was inhibited compared to control.

**FIGURE LEGENDS**

**Supplemental Figure S1**. Demonstration of purity of subcellular fractions after the subcellular fractionation. Purity and consistency of cellular fractions obtained using HUVEC lysates (T-cad, si-T-cad and control cells) was tested by Western blotting. **a** Antibody against early endosomal proteins EEA1was used to confirm that the membrane and cytosolic fractions contained the proteins of early endosomes, while nuclear fraction didn’t. **b** To prove that the membrane and nuclear fractions were free from the lysosome proteins, we used antibodies against lysosomal marker LAMP1. There are results of three independent representative experiments.

**Supplemental Figure S2**. T-cadherin doesn’t affect expression of N-cadherin or occluding, claudin-5 and ZO-1 in HUVECs and doesn’t involve VE-cadherin internalization via caveolin-dependent pathway. **a** Expression and membrane localization of N-cadherin was analyzed by Western blotting using specific anti-N-cadherin antibodies. To compare N-cadherin content on the membrane and in the cytoplasm we detected the amount of N-cadherin in total HUVEC lysates and in lysates of HUVECs after incubation in trypsin/EDTA at 37^0^C. **b** Expression of occluding, claudin-5 and ZO-1 were analyzed by Western blotting. Obtained data were normalized by GAPDH level. There are results of three independent representative experiments. **c** The confocal high-resolution images of double immunofluorescent staining of HUVECs (T-cad, si-T-cad and control cells) with antibodies against caveolin-1 (red) and VE-cadherin (green) at equal gain and offset settings. Nuclei appear blue after DAPI staining. Bar 10 µm. There are results of three independent representative experiments.

**Supplemental Figure S3.** T-cadherin induced cytoskeleton rearrangement in human endothelial cells. The confocal high-resolution images of HUVEC (T-cad, si-T-cad or control) immunostained with the antibodies against ROCK-II (red fluorescence) and phalloidin (to reveal filamentous actin) (green fluorescence) at equal gain and offset settings. **a** In control actin was distributed in the zig-zag pattern. T-cadherin overexpression resulted in the cell shape modification and the induced stress fiber formation, characterized by the deposits of thick actin bundles. T-cad cells were characterized by pronounced ROCK-II staining compared to control. In contrast to control and T-cad cells, ROCK-II expression and actin stress fiber formation in si-T-cad cells were inhibited. **b** Control, T-cad and si-T-cad cells were preincubated with Y27632, ROCK-II inhibitor, at the final concentration of 10 µM for 12 h prior to immunofluorescent staining. Application of Y27632 induced relaxation of the stress fibers in cont, T-cad and si-T-cad cells, suggesting that ROCK-II acts downstream of RhoA and mediates T-cadherin effects on the actin cytoskeleton. Nuclei appear blue after DAPI staining. Bar 25 µm. There are results of three independent representative experiments.

**Supplemental Figure S4.** T-cadherin expression doesn’t affect phosphorylation of p38, ERK1+2 or Src in HUVECs. **a** Phosphorylation of p38 on threonine (T180) and on tyrosine (Y182) was analyzed by Western blotting of total cell lysates of HUVECs (T-cad, si-T-cad and control cells) using specific anti-p-p38 antibodies against T180 and Y182. Data were normalized by total p38 content. **b** Phosphorylation of ERK1/2 on threonines (T185) and (T202) was analyzed by Western blotting of total cell lysates (T-cad, si-T-cad and control cells) using specific anti-p-ERK1+2 antibodies. Data was normalized by the total ERK1/2 content. **c** Phosphorylation of Src on tyrosine Y418 was analyzed by Western blotting of total cell lysates using specific anti-p-Src antibodies. Data was normalized by the total Src content. There was no significant difference in the level of phosphorylation of p38, ERK1/2 or Src upon the change in T-cadherin expression in HUVEC. There are results of three independent representative experiments.
